# Practical Notes and Formulæ

**Published:** 1882-04-20

**Authors:** 


### PRACTICAL NOTES AND FORMULÆ.

**Rheumatism, Acute and Chronic, Hemicrania, Etc.**—

℞. Guarana .................................. grs. xv.
With hot water, cream and sugar for a dose, and increase to forty grains once or twice a day.

Said to be almost a specific in Acute Rheumatism, and very beneficial in the cases above named.

THE CYANIDES IN ACUTE RHEUMATISM.

Dr. A. Luton, gives the Cyanide of Zinc in pill, in doses of from three-fourths to one and a half grains in a single day.

The Cyanide of Potassium, pure and well prepared, is perhaps to be preferred, he thinks, to the Salt of Zinc, on account of its evident activity. In mixture he gives it in the dose of one and a half grains per day. It is best administered in the form of pills, coated with silver. It is not advisable to go beyond two grains a day.—*Independent Practitioner.* [Give with caution!—ED. REC.]

**Iodoform in Gynecological Practice.**—Professor Bandl, of Vienna, uses iodoform in every possible variety of chronic pelvic-peritonitis, and with the most satisfactory results. It is used in emulsion with glycerine (1 to 10), and is introduced in the vagina in small quantity on a cotton tampon, where it is allowed to remain from 12 to 24 hours.

In Carl Braun's clinic, iodoform was used experimentally in acute post partum perimetritis with enormous effusion. The abdomen was painted with glycerine emulsion, and the effect was excellent, the effusion being rapidly absorbed. He ascribes the effect partly to the inhalation of the iodine vapor.—Translated by Ralph D'Ary, M. D., for the *Physician and Surgeon.*—*Western Medical Reporter.*

**Leucorrhœa.**—

℞. Zinci oxidi vel bismuth carb ............ grs. lxxx.
Ext. belladonna ......................... grs. xl.
Olei theobromæ ......................... ℥ j.;
Olei olivea ............................ ʒ iij.

Mix.—Divide into eight pessaries, and order one to be used every night.—*Medical Gazette.*

**Where the Menstrual Flow is Scanty and the Liver Sluggish.**—

℞. Podophylli resinæ ...................... grs. iv.
Ext. hyoscyami ......................... grs. xxvi.
Ext. nucis vomicæ ...................... grs. iv.
Pil. aloes et myrrhæ .................... grs. xxx.

M.—Divide into twelve pills, one to be taken at bedtime three or four nights in succession.—*Ibid.*

**Huxley's Theory of Disease.**—The body is a machine of the nature of an army, not that of a watch or of a hydraulic apparatus. Of this army, each cell is a soldier, an organ, a brigade, the central nervous system headquarters and field telegraph, the alimentary and circulatory system the commissariat. Losses are made good by recruits born in camp, and the life of the individual is a campaign, conducted successfully for a number of years, but with certain defeat in the long run. The efficacy of an army, at any given moment, depends on the health of the individual soldier, and on the perfection of the machinery by which he is led and brought into action at the proper time; and, therefore, if the analogy holds good, there can be only two kinds of diseases, the one dependent on abnormal states of the physiological units, the other on pertubation of their co-ordinating and alimentative machinery.—*Canadian Medical Record.*

**Antiseptic Stimulation in Typhoid.**—Prof. Bouchard (*Le Course. Med.*, May 7th) recommends that stimulants used in typhoid fever should be mixed with antiseptic substances, in order to prevent purulent infection from the intestinal lesions. He uses the following:

| | | | |
|---|---|---|---|
| Rum | fl. ℥ ix; | 270.00 | fl. Gm.; |
| Creosote | gtt. ij; | 0.12 | " |
| Phenic acid | gr. iv; | 0.24 | Gm.; |
| Salicylic acid | gr. xv; | 1.00 | " |

M. Sig. Small quantities of this or a similar antiseptic stimulant may be given as required.—*Mich. Med. News.*

**A Dangerous Instrument.**—According to the *Phil. Med. and Surg. Reporter*, a number of high authorities in ophthalmology have called attention to a new instrument called the "eye-cup," which has been imported from France. It is constructed on the principle of an ordinary rubber cupping-glass, but made so as to accurately fit over the eye. By pressing the rubber bulb, and then applying it, the eye is drawn out more or less by suction from its socket. It is claimed that it will relieve the presbyopia of old people, and thus render the use of glasses unnecessary. It has really been known to produce retinal congestion and hemorrhage, as well as lenticular, corneal, conjunctival, and palpebral changes, and in one case total blindness from retinal detachment was the result.—*New England Medical Gazette.*

**Application in Inflamed Conjunctiva.**—A correspondent of the Louisville Medical News, describing a visit to the Manhattan Eye and Ear Hospital, New York, supplies the formula of a solution in very common use there for inflamed conjunctiva; it is used with an atomizer in the form of spray:

| | |
|---|---|
| Tannin | grs. x. |
| Sodæ bicarb | grs xx. |
| Glycerin | ʒ ij. |
| Aquæ | Oij. |

**Treatment of Phthisical Cough.**—Mr. T. Garrett Horder (British Med. Journal,) strongly advises hydrobromic acid in doses of twenty minims. It may be given with the addition of spirits of chloroform. He has also found the inhalation of the vapor of iodine very useful in chronic cough.

Another correspondent recommends fifteen minims of hydrobromic acid and ten minims of chloric ether in a dessertspoonful of water four or five times a day, with a pill containing a quarter of a grain of codœia three times a day.

Mr. A. de Wihter Baker (Dawlish) recommends the following formula:—

| | | |
|---|---|---|
| R. | Tincturæ pruni Virginianæ | ʒ j. |
| | Glycerini | ʒ ss. |
| | Nepenthe, (Ferris and Co's.) | *m* v. |
| | Aquæ | q s. |

M. He generally orders it to be given when the cough is troublesome, and repeated in three or four hours, if required. In troublesome cases he also orders a double dose to be given at bedtime. He has never known it to fail to relieve cough; and it can be taken for a long period of time without disturbing the digestive organs.—*Med. & Surg. Reporter.*

**Hair Tonic.**—J. M. Frey, in Medical Brief, says he has used the following successfully for falling hair after fevers:—

| | |
|---|---|
| Zinci sulph | 10 grs. |
| Quiniæ sulph | 20 grs. |
| Tinct. cantharides | 1 oz. |
| Bay rum | 2 oz. |
| Glycerinæ | 2 oz. |
| Water | 2 oz. |

M. To be brushed or rubbed into the scalp with much gentle shampooing.—*Jour. Chemistry.*

**Borax in Hoarseness.**—This salt has been employed with advantage in cases of hoarseness and aphonia occurring suddenly from the action of cold. The remedy is recommended to singers and orators whose voices suddenly become lost, but which by these means can be recovered almost instantly. A little piece of borax, the size of a pea, is to be slowly dissolved in the mouth ten minutes before singing or speaking. The remedy provokes an abundant secretion of saliva, which moistens the mouth and throat. This local action of the borax should be aided by an equal dose of potassium, taken in warm solution before going to bed.—*La France Medicale.*

**Treatment of Malarial Chill.**—At the Bellevue Hospital the following means are, among others, employed to prevent malarial chill. 1. The hypodermic injection of pilocarpine, gr. 1-6. 2. The inhalation of gtt. v. of amyl nitrite every twenty to thirty minutes. 3. The administration of chloroform and whisky, of each ℥ ss. The excessive diarrhœa of typhoide is said to be remarkably controlled by the administration of gtt. xx. of turpentine every two or three hours.—*Medical Record.*

